# Efficacy and safety of peripheral magnetic stimulation for the treatment of intractable hiccups after stroke: a prospective, blinded, parallel randomized controlled clinical trial

**DOI:** 10.3389/fneur.2025.1615318

**Published:** 2025-08-13

**Authors:** Qiliang Liu, Yijia Jiang, Jingbo Sun, Huiyu Liu, Junbin Chen, Chenze Jiao, Daiyi Chen, Zicai Liu

**Affiliations:** ^1^Guangzhou University of Traditional Chinese Medicine, Guangzhou, China; ^2^Yue Bei People’s Hospital, Shaoguan, China; ^3^Department of Rehabilitation Medicine, Foresea Life Insurance Shaoguan Hospital, Shaoguan, China; ^4^Department of Rehabilitation Medicine, Shaoguan First People’s Hospital, Shaoguan, China

**Keywords:** magnetic stimulation, intractable hiccups, stroke, RCT, rTMS

## Abstract

**Aims:**

Preclinical studies indicate that magnetic stimulation may be an efficacious treatment for intractable hiccups in post-stroke patients. This study aimed to investigate repetitive peripheral magnetic stimulation (rPMS) potential efficacy and safety for treating intractable hiccups.

**Methods:**

This randomized controlled trial randomly assigned 60 patients with stroke with intractable hiccups to receive rPMS (*n* = 30) or metoclopramide (*n* = 30). The control group received a 10 mg metoclopramide injection intramuscularly twice daily, while the experimental group underwent daily repeated magnetic stimulation at 5 Hz with 1,200 stimuli using a round coil transversally positioned below the xiphoid process. Metoclopramide or rPMS was administered until the hiccups were entirely ceased. The efficacy of the two groups was evaluated after 1 week of treatment.

**Results:**

All 60 enrolled male patients completed the study. The proportion of patients achieving complete cure was significantly higher in the magnetic stimulation group than in the metoclopramide group (23/30 vs. 15/30; *p* = 0.032). However, total response rate (cure + improvement) did not differ significantly between groups (29/30 vs. 28/30; *p* = 1.000). No significant differences were observed in recurrence rates (*p* = 0.052). Just one case of fatigue and one case of dizziness were observed in the metoclopramide group.

**Conclusion:**

Magnetic stimulation may be superior to metoclopramide in achieving complete cure of intractable hiccups after stroke, though both treatments show high overall response rates.

**Clinical trial registration:**

https://www.chictr.org.cn/, ChiCTR2200060435.

## 1 Introduction

Hiccups, medically termed diaphragmatic spasms, occur when an abrupt inhalation is followed by rapid glottis closure, producing the characteristic sound “ic” due to stimulation of the phrenic nerve, vagus nerve, or central nervous system (1). Hiccups that persist for >48 h and cannot be alleviated spontaneously are clinically diagnosed as intractable hiccups (2). Intractable hiccups often manifest as a symptom of various diseases, primarily associated with disorders of the central nervous system and the gastrointestinal tract (1, 3). Central hiccups following a stroke are common complications (4) that can affect the eating, breathing, sleep, and daily life of patients. Intractable hiccup often occurs in the early stage of acute cerebrovascular disease. No official guidelines exist for the therapy of intractable hiccups. The **management** is based on prior experience and anecdotal evidence, and frequently involves old medications (5). Metoclopramide (a dopamine D_2_ receptor antagonist) (6, 7), chlorpromazine (a dopaminergic antagonist) (8, 9), baclofen (a GABAₐ agonist) (10, 11), and gabapentin (a GABA analog) (12, 13) are commonly used in clinical treatment. Most medications for hiccups act through dopaminergic or GABAergic pathways (14); Metoclopramide was selected as the active comparator in this trial due to its widespread clinical use, parenteral formulation suitability for controlled administration, and documented efficacy in prior hiccup studies (6, 7). However, their efficacy is limited, and some patients may develop adverse reactions. Physical therapy for hiccups primarily involves acupuncture (15, 16) and electrical stimulation (17, 18).

A clinical case report demonstrated that magnetic stimulation is a workable physical therapy for intractable hiccups, specifically in patients with stroke (19). Repetitive peripheral magnetic stimulation (rPMS) was used to treat three cases of intractable hiccups after stroke in the clinical pre-experiment, all of which were resolved within 1 week without adverse reactions. The study on rPMS for hiccups is in its early stages and requires further advancement. However, the small sample size and the absence of a conventional control group hinder assessing whether the therapeutic effect surpasses that of conventional treatment. Therefore, a randomized, controlled clinical study on rPMS for intractable hiccups after stroke is imperative.

## 2 Materials and methods

### 2.1 Study design

This randomized controlled trial enrolled 60 patients with intractable hiccups after stroke from the Department of Neurology of Yuebei People’s Hospital between June 2022 and September 2024. The patients were randomly divided into two groups, with 30 patients in each group. Patients in the experimental group received magnetic stimulation once daily, while those in the control group received metoclopramide injections. This clinical investigation was granted by the Medical Ethics Committee of Yuebei People’s Hospital (KY-2022-013), conducted by the Declaration of Helsinki, and registered at https://www.chictr.org.cn/ (ChiCTR2200060435). The efficacy and adverse reactions were assessed after 1 week of treatment. A follow-up was conducted after 1 month to determine if the hiccup reoccurred. This study was supported by the Shaoguan City Health and Wellness Research Project (No. Y24048).

### 2.2 Inclusion and exclusion criteria

The inclusion criteria required patients who met the diagnostic criteria for intractable hiccups after stroke (20); patients between the ages of 40 and 80 years with intractable hiccups (>48 h) after stroke; patients who provided written informed consent. The exclusion criteria were as follow: patients with other causes of hiccups, including encephalitis, multiple sclerosis, epilepsy, Parkinson’s syndrome, neuromyelitis optica, traumatic brain injury, gastrointestinal diseases, myocardial ischemia, kidney failure, pneumonia, bronchitis, laryngitis, and chemotherapy (1, 3); patients with mental illness; pregnant or lactating women; Patients with cardiac pacemakers, epilepsy and other diseases are not suitable for magnetic stimulation; patients participating in other clinical trials.

### 2.3 Randomization

A random number table was used to generate random numbers, which were placed in a numbered opaque envelope and sealed. Corresponding envelopes were opened according to the treatment sequence.

### 2.4 Participant recruitment and screening

This study recruited all patients with stroke who had been admitted to Yuebei People’s Hospital. We administered metoclopramide or magnetic stimulation treatment to 60 patients with stroke between June 2022 and September 2024. This enabled a fair evaluation of the feasibility criteria, which permitted a precise calculation of the efficacy of the magnetic stimulation for calculating the subsequent sample size. We informed the patients enrolled to receive metoclopramide or magnetic treatment about the study and gave them an information sheet. The patients signed an informed consent form to participate in this study. After the clinical assessment, the patients were randomized to receive either metoclopramide or magnetic stimulation, which was administered by trained therapists. All eligible stroke patients (regardless of sex) were invited.

### 2.5 Intervention plan

**Figure 1:**
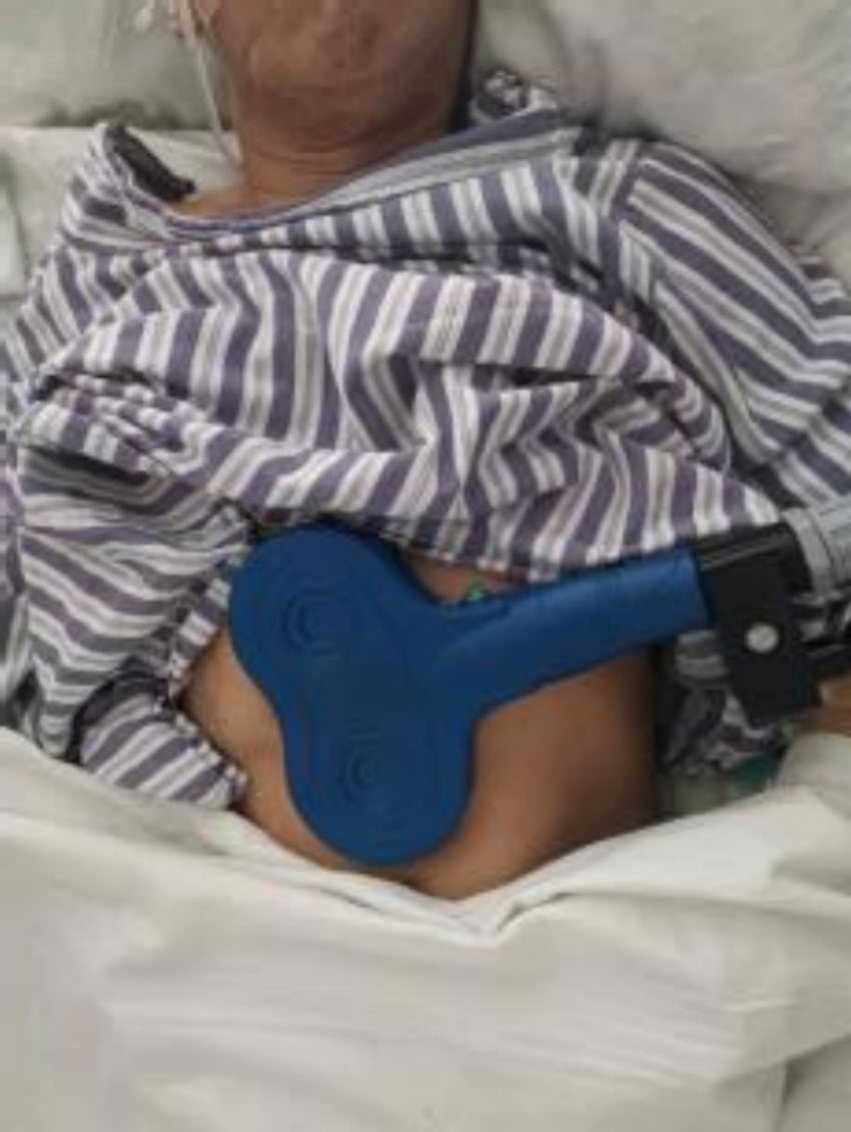
Treatment of details.

Patients in the metoclopramide (control) group received 10 mg of metoclopramide via intramuscular injection twice daily for 7 days until hiccups ceased. Patients in the experimental group received magnetic stimulation with a pulse frequency of 5 Hz. Each pulse train lasted for 2 s, consisting of 120 trains of 10 pulses interspersed with 6 s intervals at a tolerable intensity (55% of the RMT). We refer to previous studies of TMS for the treatment of hiccups, including 1 Hz and 10 Hz (19, 21), and there are no studies of direct stimulation of the diaphragm with a magnetically stimulated figure-of-eight coil to which we can refer. In our pre-experiment, a frequency of 5 HZ, which is moderate between the two, was taken and formulated concerning the number of pulses of other similar peripheral magnetic stimulation and conventional treatments1200 (22–24), which was discussed many times by the team. A round coil was transversally positioned below the xiphoid process (Figure 1). The patients underwent magnetic stimulation sessions once daily for 7 days until hiccups ceased. The magnetic field stimulator was manufactured by Wuhan Ired Medical Equipment New Technology Co. Ltd. The model is YRD CCY-1.

### 2.6 Assessments

The patients’ outcomes were evaluated following the standard criteria established by the Chinese Medicine Medical Association and previous study findings (16). They are classified as follows:

**Cure**, the symptoms of hiccups entirely resolved within 7 days.

Improvement: ≥50% reduction in hiccup frequency or intensity (e.g., from persistent to occasional episodes) within 7 days, but not complete resolution.

**No effect**, no resolution of hiccups.

**Recurrence**, Return of hiccups (≥1 episode/day) within 1 month after initial cure.

### 2.7 Data collection

The efficacy and adverse reactions were recorded in both groups based on clinical outcome. we collected data on 60 stroke patients with intractable hiccups willing to be randomized and complied with the intervention. Outcome assessors and statisticians were blinded; therapists/patients were unblinded due to intervention nature. Metoclopramide group received injections without sham rPMS.

### 2.8 Sample size estimation

According to the results of the previous exploratory study and the findings from relevant references, a non-inferiority proportion comparison was employed. The sample size estimation formula was designed, so the following formula was used for estimation: $n=(\frac{p_{A}(1-p_{A})}{k}+p_{B}(1-p_{B}))(\frac{z_{1-\alpha }+z_{1-\beta }}{p_{A}-p_{B}-\delta })$ , Among them, *n* is the sample size, α is the test level, β is the type II error, 1-β is the power of confidence, α = 0.05, β = 0.2, $p_{A}$ and $p_{B}$ are the effective rates of the experimental group and the control group, respectively 85 and 65% (7, 19), *k* is the sample ratio, *k* = 1, δ is the non-inferiority margin, δ = −0.1, It was calculated that *n* = 25. Considering the dropout of 10%, finally, the sample size of each group was determined to be 30 cases.

### 2.9 Data analysis

The preliminary analysis tested the baseline data of patients randomized for the trial. The Fisher exact or Chi-square test was used to assess the differences between groups in categorical data. The *t*-test or Mann–Whitney U-test was used for continuous data. Additionally, relative risks and 95% confidence intervals were reported. A *p*-value < 0.05 was considered statistically significant. Analysis of data was performed by a statistician who was anonymized to the study group. IBM SPSS 24.0 was used for all statistical analyses.

## 3 Results

**Figure 2:**
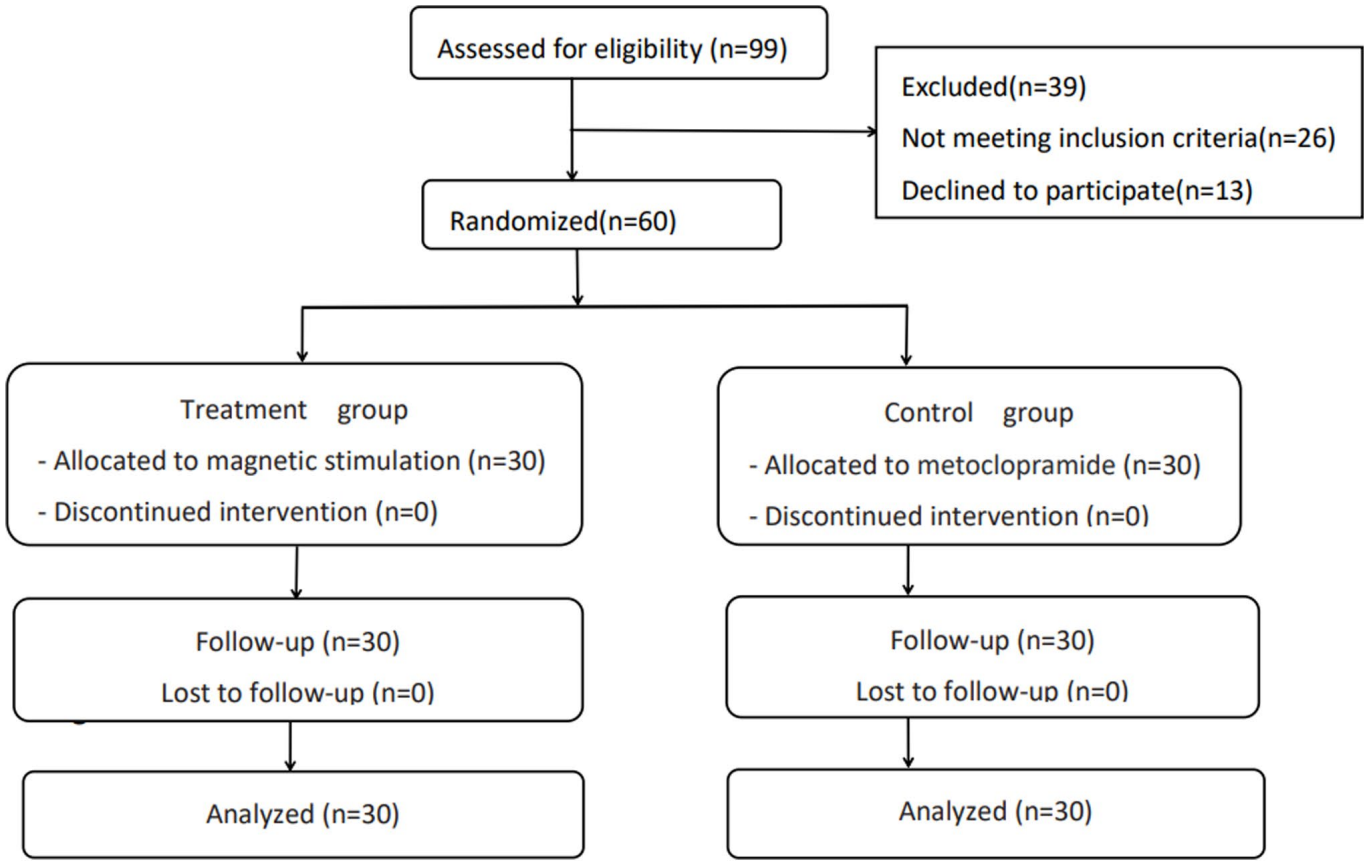
Flow chart of participants through the trial.

Herein, 99 patients were screened for eligibility. Of those 99 (66 male/33 female) patients, 26 (6 male/20 female) did not meet the inclusion criteria, and 13 female patients who met the inclusion criteria refused to participate. Therefore, 60 patients were randomly included in the final analysis (Figure 2). Baseline characteristics revealed no significant differences between the two groups on most sociodemographic and clinical variables (Table 1). The mean age of patients in the magnetic stimulation group was 62.23 ± 8.13 years, while the mean age of patients in the metoclopramide group was 62.10 ± 9.46 years. In the magnetic stimulation group, 26 and 4 patients had ischemic stroke and hemorrhagic stroke, respectively, while in the metoclopramide group, 27 and 3 patients had ischemic and hemorrhagic strokes. Brain stem lesions were the most common cause of central hiccups among 15 patients in each group, respectively. Additionally, massive hemispheric infarction was one of the leading causes of central hiccups among 6 and 7 patients in the metoclopramide and magnetic stimulation groups. Due to the higher incidence of hiccups in men at screening and the fact that all of the only 13 women declined, only the male cohort was available.

**Table 1:** Baseline information of subjects.

Baseline indicators	Variable	Group		*p* value
		Metoclopramide (*n* = 30)	Magnetic stimulation (*n* = 30)	
Age	Mean (SD)	62.10 (9.46)	62.23 (8.13)	0.954
Gender	Male	30 (100.00%)	30 (100.00%)	1.000
	Female	0 (0.00%)	0 (0.00%)	1.000
Married		28 (93.33%)	30 (100.00%)	0.492
Ethnicity	Han Chinese	30 (100.00%)	30 (100.00%)	1.000
Duration of hiccups (days)	M (*P*_25_, *P*_75_)	2 (2, 2.25)	2 (2, 3)	0.190
Duration of stroke (days)	M (*P*_25_, *P*_75_)	6 (2.75, 8.75)	5 (3, 9)	0.823
Severity of stroke (NIHSS)	M (*P*_25_, *P*_75_)	7.5 (1.75, 15.5)	8 (5, 16.75)	0.428
Stroke	Ischemic	27 (93.33%)	26 (86.67%)	1.000
	Hemorrhagic	3 (6.67%)	4 (13.33%)	1.000
Lesion location	Brainstem	15 (50.00%)	15 (50.00%)	1.000
	Massive hemispheric infarction	6 (20.00%)	7 (23.33%)	0.754
	Other lesion location	9 (30.00%)	8 (26.67%)	0.774

Continuous data are expressed as mean ± SD or median (interquartile range); categorical data are expressed as frequency (percentage). The *t*-test was used for age and Mann–Whitney U-test for other continuous data; the Fisher exact test was used for gender and ethnicity, Chi-square test for other categorical data. Chi-square test was used for comparison of clinical assessments.

After the intervention, as shown in Table 2, After the intervention, complete cure was achieved in 23 patients (76.7%) in the magnetic stimulation group versus 15 (50.0%) in the metoclopramide group (95% CI, 1.085–9.952; *p* = 0.032). Symptomatic improvement (without full cure) occurred in 6 patients (20.0%) with magnetic stimulation versus 13 (43.3%) with metoclopramide (95% CI, 0.104–1.032; *p* = 0.052). One patient in the magnetic group had no effect of the hiccups, compared with 2 patients in the metoclopramide group (95% CI, 0.041–5.628; *p* = 1.000). One patient in the magnetic stimulation group relapsed, compared to 2 patients in the metoclopramide group (95% CI, 0.041–5.628; *p* = 1.000). In addition, a significant difference in complete cure rate was found (*p* < 0.05), while total response rate (cure + improvement) did not differ significantly (96.7% vs. 93.3%; *p* = 1.000; Table 2).

**Table 2:** Comparison of clinical assessments between the two groups.

Outcomes	Magnetic stimulation (*n* = 30)	Metoclopramide (*n* = 30)	Relative risk (95% CI)	*p* value
Cure	23 (76.7%)	15 (50%)	3.286 (1.085–9.952)	0.032
Improvement	6 (20%)	13 (43.3%)	0.327 (0.104–1.032)	0.052
No effect	1 (3.3%)	2 (6.7%)	0.483 (0.041–5.628)	1.000
Recurrence	1	2	0.483 (0.041–5.628)	1.000
Total response	29 (96.7%)	28 (93.3%)	0.483 (0.041–5.628)	1.000

Recurrence was assessed only in patients initially cured (*n* = 38). Total response = Cure + Improvement.

No severe adverse events requiring discontinuation occurred. Tolerability data were limited to spontaneously reported events: one case of transient fatigue (resolved within 2 h without intervention) and one case of mild dizziness (resolved after 30-min rest) in the metoclopramide group. No discomfort or treatment interruption was reported in the rPMS group, though formal assessment of subjective tolerance (e.g., visual analog scales) was not conducted.

## 4 Discussion

In terms of etiology, hiccups can be broadly divided into central and peripheral types (25). Intractable central hiccups are one of the common symptoms in patients with posterior circulation stroke (26). Magnetic stimulation achieved a significantly higher rate of complete cure (76.7% vs. 50.0%, *p* = 0.032) compared to metoclopramide, suggesting superior efficacy in achieving total symptom resolution. However, the similar total response rates (96.7% vs. 93.3%, *p* = 1.000) indicate that both interventions effectively reduce symptoms, albeit through distinct mechanisms. For patients with intractable hiccups, complete cessation may represent a more clinically meaningful endpoint than partial improvement. The final 60 patients included in this study were all male, as the 19 female patients who were previously excluded from the trial either did not meet the inclusion criteria or declined to participate in the study. This study observed in this cohort that men are more susceptible to hiccups, consistent with current reports (3, 25, 27). However, this finding is inconsistent with previous research, which reported that the gender difference in hiccups was more significant in patients with non-CNS causes; conversely, it was unclear in patients with CNS causes (25). Previous studies reported that male susceptibility to hiccups may be related to lower synaptic thresholds and increased excitability of afferent or efferent nerves in the hiccup reflex arc among males (28, 29).

This study suggested that the predisposition for males to develop hiccups is associated with the central nervous system rather than the peripheral nervous system. Medullary infarction is a primary cause of central hiccups. We identified 22 female patients with medullary infarction during the study period, none of whom developed central hiccups, and 10 vomited. However, among 38 male patients with medullary infarction, 11 experienced central hiccups, and 9 vomited. It is hypothesized that the hiccup and vomiting center in the medulla is activated after a stroke. Men are more susceptible to hiccups, and women are more vulnerable to vomiting. Gender difference was also observed between chemotherapy-induced vomiting and hiccups, with men exhibiting a significantly higher incidence of chemotherapy-induced hiccups while women exhibited a significantly higher rate of emesis and nausea (30). The mechanisms underlying this gender discrepancy require investigation.

Although there are many ways to treat hiccups, a Cochrane systematic review reported that there is insufficient evidence to guide pharmacologic and nonpharmacologic treatment of intractable hiccups (31). Three physical methods for alleviating hiccups by suppressing or interrupting the hiccup reflex arc are nasopharyngeal stimulation, vagal stimulation, and respiratory maneuvers (1). However, these maneuvers are effective only for alleviating acute hiccups and not for intractable hiccups (1). Magnetic stimulation is widely used in cerebral cortex functional area stimulation therapy, and it can stimulate human body tissue non-invasively and painlessly. It includes central repetitive transcranial magnetic stimulation (32, 33) and rPMS (24); however, rPMS was less commonly reported. rPMS can be used for stroke rehabilitation (34, 35) and various pain disorders (36, 37). This study demonstrated that rPMS is a simple, specific, and harmless method. It has advantages over traditional electrical stimulation, including the external diaphragm pacemaker. Magnetic stimulation therapy can stimulate peripheral nerves and muscles, utilizing a circular coil during high-frequency stimulation. Because the circular coil has a large action area, producing temporal and spatial superposition of muscle stimulation is easy. It can achieve a stronger stimulation effect than electrical stimulation. Electrical stimulation has a weak penetration force and is difficult to reach deep muscles, while magnetic stimulation has comprehensive coverage and intense penetration (38). While the precise neurophysiological mechanisms underlying rPMS efficacy in hiccup suppression require further investigation, our findings align with the hypothesis that high-frequency stimulation modulates the hiccup reflex arc. rPMS applied below the xiphoid process likely targets the phrenic nerve terminals and diaphragmatic muscle fibers, inducing sustained contractions that may disrupt aberrant phrenic nerve signaling through superstimulation-induced fatigue (39). This peripheral modulation could interrupt the afferent limb of the hiccup reflex arc, which involves phrenic/vagal inputs to medullary centers (1). Furthermore, magnetic fields penetrate deeper tissues than electrical stimulation (40), potentially influencing medullary nuclei involved in hiccup generation (e.g., nucleus tractus solitarius) via trans-synaptic effects (41). The observed efficacy supports prior case reports of magnetic stimulation normalizing diaphragmatic rhythm in intractable hiccups (19), though direct neurophysiological evidence (e.g., EMG studies of diaphragmatic entrainment) remains limited and warrants future study.

This study also has some limitations, such as sample Generalizability and Gender considerations significant limitation of the present study is the exclusive enrollment of male participants. While this occurred due to the higher incidence of hiccups among screened male stroke patients and the refusal of all eligible female patients (*n* = 13) to participate (resulting in no female cases available for randomization), it introduces potential selection bias. Consequently, our findings regarding the efficacy and safety of rPMS compared to metoclopramide are strictly applicable only to the male post-stroke population with intractable hiccups. We explicitly acknowledge this limitation and emphasize that the generalizability of our results to female patients is currently unknown and cannot be inferred from this study. Future research must prioritize the inclusion of female participants to assess potential gender differences in treatment response and establish the broader applicability of rPMS for intractable hiccups. Gender differences were not a primary aim; findings are observational. It is difficult to predict the effect of magnetic stimulation due to different factors such as age, basic health status, location, and severity of stroke, etiology, and course of hiccups. Moreover, the parameters of magnetic stimulation were not investigated in this study. The parameters of magnetic stimulation, such as intensity, frequency, and stimulation time, need to be adjusted according to the specific conditions of the patient. However, there are no uniform standard parameters, which may lead to compromised treatment effects. Future clinical studies with large samples should be conducted to determine the therapeutic dose of magnetic stimulation based on the analysis. Fifth, while we recorded major adverse events, systematic assessment of tolerability (e.g., treatment-related discomfort scales) and patient acceptability (e.g., satisfaction surveys) was not performed. This limits our understanding of the intervention’s feasibility in real-world settings. Future trials should incorporate patient-reported outcomes to comprehensively evaluate therapeutic utility.

## 5 Conclusion

This is the first randomized controlled study of 5 Hz rPMS for the treatment of hiccups. This pilot study demonstrated that magnetic stimulation has significant potential in treating intractable hiccups, though its clinical applicability is constrained by unmeasured tolerability and acceptability parameters. Future large-scale trials should integrate patient-centered outcomes.

## Data Availability

The original contributions presented in the study are included in the article/supplementary material, further inquiries can be directed to the corresponding author.
